# Microbiome in women with endometriosis and the *in vitro* effects of *Lactobacillus reuteri* on human endometrium

**DOI:** 10.1128/spectrum.03689-25

**Published:** 2026-05-06

**Authors:** Jae Hoon Lee, Gee Soo Jung, Kyungmin Kim, Hyemin Park, Yunjeong Park, Inha Lee, Min Jung Lee, Ji-Ho Lee, Young Sik Choi, SiHyun Cho

**Affiliations:** 1 Department of Obstetrics and Gynecology, Division of Reproductive Endocrinology, Gangnam Severance Hospital, Yonsei University College of Medicine 37991, Gangnam-gu, South Korea; 2 Department of Integrative Medicine, Yonsei University College of Medicine 37991, Seodaemun-gu, South Korea; 3 Department of Obstetrics and Gynecology, Division of Reproductive Endocrinology, Severance Hospital, Yonsei University College of Medicine 37991, Seodaemun-gu, South Korea; 4 School of Natural Resources and Environment Science, College of Agriculture and Life Sciences, Kangwon National University 98410, Chuncheon-si, South Korea; Nova Southeastern University, Fort Lauderdale, Florida, USA

**Keywords:** endometriosis, microbiome, *Lactobacillus reuteri*, estrogen metabolism, *in vitro *co-culture, β-glucuronidase

## Abstract

Endometriosis (EMS) is a chronic inflammatory disorder affecting ~10% of reproductive-age women, with increasing evidence implicating the microbiome in its pathogenesis through immunomodulation and estrogen metabolism. This study investigated microbiome composition in the vagina, endometrium, and peritoneal fluid (PF) of women with and without EMS and further assessed the effects of *Lactobacillus reuteri* (*L. reuteri*) on endometrial (EM) cells *in vitro*. Samples from 41 patients were analyzed using 16S rRNA gene sequencing, targeting the V3–V4 regions. Western blotting, ELISA, and LC-MS/MS were employed to evaluate protein expression and estrogen metabolism during EM–*L*. *reuteri* co-culture with or without estradiol-17-glucuronide (E2G). Microbiome analysis revealed no significant differences in alpha or beta diversity between EMS and controls across all compartments. However, LEfSe analysis identified several taxa with differential abundance, with *L. reuteri* consistently altered in both vagina and EM. Across the menstrual cycle, EM and vaginal microbiomes were stable, whereas PF microbiota showed phase-dependent variation involving 60 genera and 76 species. *In vitro*, *L. reuteri* alone did not alter endometriosis-related proteins, but in the presence of E2G, it reduced BAX/Bcl-2 ratios and increased p-NF-κB, suggesting anti-apoptotic and pro-inflammatory shifts. Progesterone receptor α/β expression decreased, while estrogen receptor levels remained unchanged. *L. reuteri* increased β-glucuronidase activity but did not enhance E2G-to-estradiol conversion. These findings highlight *L. reuteri* as a potentially important species in EMS, with *in vitro* evidence suggesting survival-promoting effects under estrogenic conditions. Further research should explore multi-species interactions and hormonal contexts to clarify microbial contributions to EMS pathogenesis.

**IMPORTANCE:**

Although *Lactobacillus reuteri* appeared more abundant in the vagina and endometrium of controls, suggesting a protective role, *in vitro* findings paradoxically indicated anti-apoptotic and pro-inflammatory effects under estrogenic conditions, underscoring the need for further investigation of multi-species microbial interactions and hormonal contexts in endometriosis pathogenesis.

## INTRODUCTION

Endometriosis (EMS) is an inflammatory condition characterized by the abnormal growth of endometrial (EM) tissue outside the uterus, impacting approximately 10% women of reproductive age globally (1, 2). EMS commonly infiltrates pelvic organs such as the ovaries and pelvic peritoneum; however, implants can also be found throughout the abdomen, such as on the bladder and bowel, resulting in painful symptoms such as dysmenorrhea, chronic pelvic pain, and dyspareunia (3). EMS is commonly attributed to the reflux of menstrual blood into the pelvis via the fallopian tubes. Considering that endometriosis affects only approximately 10% of women, whereas retrograde menstruation of EM tissue mixed with blood is routinely observed in nearly all ovulatory women (4), it is improbable that this phenomenon is the sole cause of the disease. While this reflux is believed to contribute to the development of EMS, the precise factors influencing its onset remain unclear.

Recent studies have shown a strong link between the human microbiome and various diseases, including EMS. The microbiome contributes to physiological homeostasis by regulating immune and inflammatory processes (5, 6), and influencing estrogen metabolism, which in turn affects microbial composition (7). β-glucuronidase produced by certain microbes can deconjugate conjugated estrogens, enhancing estrogenic activity, and may also contribute to inflammation by modulating immune cell function (6, 8). Given that EMS is an estrogen-dependent inflammatory disorder, these hormone–microbiome interactions are likely to play a crucial role in its development and progression.

Previous studies have identified microbial dysbiosis in the reproductive tract and gut of women with EMS, but most have focused on compositional rather than functional differences. Notably, Muraoka et al. showed that Fusobacterium infection activates TGF-β signaling in EM cells, inducing fibroblast-to-myofibroblast transition (9), while Wei et al. demonstrated that elevated β-glucuronidase expression promotes EMS progression by impairing macrophage function *in vivo* (10). These findings suggest that specific microbes can directly modulate molecular and cellular pathways involved in EMS pathogenesis, although the broader functional interactions between microbial activity and disease mechanisms remain largely unexplored.

In this study, we analyzed the microbiome composition of the reproductive tract—the vagina, endometrium, and pelvic peritoneum—in women with and without EMS using 16S rRNA gene sequencing. Beyond compositional profiling, we investigated the functional relevance of *Lactobacillus reuteri* (*L. reuteri*), which showed the greatest difference in abundance in both vaginal and EM samples of women with EMS. EM cells from women without the disease were co-cultured with *L. reuteri* to evaluate cellular and molecular responses, focusing on β-glucuronidase activity and its role in the conversion of estradiol-17-glucuronide (E2G) to estradiol. These analyses aimed to elucidate how *L. reuteri* may contribute to the cellular and biochemical processes underlying EMS pathogenesis.

## MATERIALS AND METHODS

### Microbiome analysis using next-generation sequencing (NGS) in women with endometriosis: study population and sample collection

A total of 45 premenopausal women aged 20–45 years with regular menstrual cycles were enrolled. Patients scheduled for surgery due to suspected EMS based on radiological findings (ultrasound or MRI) comprised the EMS group, while patients undergoing surgery for dermoid cysts without evidence of EMS or adenomyosis served as controls.

Women were excluded from the study if they did not receive a post-operative diagnosis of EMS or dermoid cyst, had a history of female hormone therapy, probiotics, antibiotics, or immunosuppressants within the past two months, had a history of previous abdominal surgery or pelvic inflammatory disease, or were pregnant. Additionally, women with malignant tumors, autoimmune diseases, or a BMI greater than 30 kg/m^2^ were also excluded.

Specimens were collected from the vagina and endometrium of the patient in the lithotomy position, immediately before skin preparation and prior to surgery. Vaginal and EM swabs were collected following the insertion of a sterile vaginal speculum using OMNIgene vaginal device (DNA Genotek Inc., Ottawa, Canada). Two separate swabs and collection tubes were used for vaginal and EM samples. When collecting samples from endometrium, care was taken to avoid the swab touching the cervical walls. Both samples were immediately transferred to −80°C to be stored in an upright position until DNA extraction.

To obtain peritoneal fluid (PF), the patient’s skin was prepared, a 1 cm incision was made in the umbilicus, and a Glove Port (Meditech Inframed Corp., Seoul, South Korea) was inserted through the incision. After the endoscope entered the abdominal cavity, if body fluid was present in the posterior cul-de-sac, samples were collected aseptically. If no body fluid was present, samples were not collected. A minimum of 5 mL fresh PF was collected in a 15 mL Falcon tube and rapidly transferred to −80°C to be stored in an upright position until DNA extraction. Preoperative prophylactic antibiotics were administered after sample collection.

### DNA extraction

All samples were subsequently transported on dry ice to Macrogen, Inc. (Seoul, South Korea) for DNA extraction. The DNeasy PowerSoil Pro Kit (Qiagen, Hilden, Germany) was employed for DNA extraction, with minor modifications to the manufacturer’s protocol to accommodate our specific sample types. The procedure commenced with the addition of 800 µL of CD1 solution to a PowerBead Tube, followed by the introduction of the sample. The tube was briefly vortexed to ensure proper mixing, then secured horizontally on a vortex adapter and subjected to vortexing at 1,500 rpm for 15 min. Post-vortexing, the mixture was centrifuged at 15,000 × *g* for 1 min to pellet any debris. The supernatant, approximately 500–600 µL, was carefully transferred to a clean 2 mL microcentrifuge tube, to which 200 µL of the refrigerated CD2 solution was added. After vortexing the mixture for 5 s, it was centrifuged again at 15,000 × *g* for 1 min. Without disturbing the pellet, 700 µL of the supernatant was transferred to another clean 2 mL microcentrifuge tube, followed by the addition of 600 µL of CD3 solution and another brief vortexing for 5 s. The resulting mixture was loaded onto an MB spin column in 650 µL increments, each followed by centrifugation at 15,000 × *g* for 1 min, with the flow-through being discarded after each spin until the entire sample had been processed. The column was then washed with 500 µL of EA solution, centrifuged at 15,000 × *g* for 1 min, and the flow-through discarded. This was followed by another wash with 500 µL of C5 solution, also centrifuged at 15,000 × *g* for 1 min, with the flow-through discarded. To ensure complete removal of any residual wash buffer, the column was subjected to a final centrifugation at 16,000 × *g* for 2 min. The spin column was then transferred to a new 1.5 mL Eppendorf tube, and 32 µL of C6 solution was added directly to the center of the column membrane. After incubating for at least 3 min, the tube was centrifuged at 14,000 rpm for 1 min to elute the purified DNA.

### Library construction and sequencing

Sequencing libraries were prepared following the Illumina 16S Metagenomic Sequencing Library protocols to amplify the V3 and V4 regions of the 16S rRNA gene. The input genomic DNA (gDNA) at 2 ng or 10 ng was PCR amplified using a 5× reaction buffer, 1 mM dNTP mix, 500 nM each of the universal forward and reverse PCR primers, and Herculase II fusion DNA polymerase (Agilent Technologies, Santa Clara, CA, USA). The first PCR cycling conditions were as follows: an initial heat activation at 95°C for 3 min, followed by 25 cycles of 30 s at 95°C, 30 s at 55°C, and 30 s at 72°C, with a final extension at 72°C for 5 min. The universal primer pair with Illumina adapter overhang sequences used for the first amplification was:

16S amplicon PCR forward primer

5′-TCGTCGGCAGCGTCAGATGTGTATAAGAGACAGCCTACGGGNGGCWGCAG-3′

16S amplicon PCR reverse primer

5′-GTCTCGTGGGCTCGGAGATGTGTATAAGAGACAGGACTACHVGGGTATCTAATCC-3′

The first PCR product was purified using AMPure beads (Agencourt Bioscience, Beverly, MA, USA). Following purification, 10 µL of the first PCR product was subjected to a second round of PCR for final library construction, which included indexing using Nextera XT Indexed Primers. The second PCR was performed under the same conditions as the first PCR, but with only 10 cycles. The resulting PCR product was again purified using AMPure beads. The final purified product was quantified using qPCR, following the qPCR Quantification Protocol Guide (KAPA Library Quantification Kits for Illumina Sequencing platforms), and qualified using the TapeStation D1000 ScreenTape (Agilent Technologies, Waldbronn, Germany). Sequencing was then performed using the MiSeq platform (Illumina, San Diego, USA).

### Operational taxonomic unit (OTU) analysis

After sequencing was completed, MiSeq raw data were classified by sample using the index sequence, and FASTQ files were created for each sample. The adapter sequence was removed using the fastp program (11), and error correction was performed on the region where the two reads overlapped. Paired-end data separated for each sample were assembled into one sequence using FLASH (1.2.11) (12). If the assembled sequence was less than 400 bp or greater than 500 bp in length, it was removed.

The obtained sequences were processed using CD-HIT-OTU (13), an OTU analysis program based on CD-HIT-EST, to eliminate low-quality, ambiguous, and chimeric sequences identified as potential sequencing errors. Subsequently, sequences exhibiting ≥97% similarity were clustered into OTUs at the species level.

For the representative sequence of each OTU, BLASTN (v2.9.0+) (14) was conducted against the NCBI 16S Microbial Reference database, and taxonomic assignment was based on the organism information of the subject with the highest sequence similarity. Taxonomy was not assigned if the query coverage of the best database match was below 85% or if the identity of the aligned region was less than 85%.

Comparative analyses of microbial community composition were performed using QIIME (v1.9) (15) based on the abundance and taxonomic classification of the identified OTUs. Species diversity and evenness within the microbial communities were evaluated using the Shannon Index and Inverse Simpson Index, while alpha diversity was assessed through Chao1 estimates. Beta diversity, representing differences in microbial composition among sample groups, was calculated using Weighted and Unweighted UniFrac distances. Relationships among samples were visualized using principal coordinate analysis (PCoA) and an Unweighted Pair Group Method with Arithmetic Mean (UPGMA) dendrogram (15, 16). Additionally, a Venn diagram was generated to compare the presence or absence of microorganisms across groups.

### ASV generation and taxonomic assignment

After sequencing, adapter and primer sequences were removed using Cutadapt (v3.2), and forward and reverse reads were trimmed to 250 and 200 bp, respectively (17). Amplicon sequence variants (ASVs) were generated using DADA2 (v1.18.0), which performed quality filtering, error correction, denoising, paired-end merging, and chimera removal using the consensus method. Reads with ≥2 expected errors and ASVs shorter than 350 bp were excluded (18). Data were normalized in QIIME (v1.9.0) by rarefaction to the minimum sequencing depth across samples (15). Taxonomic assignment was conducted using a Bayesian classifier against the NCBI 16S reference database with a confidence threshold of 50% (19). The resulting ASVs were used for downstream analyses.

### ANCOM-BC analysis

Differential abundance between control and EMS groups was evaluated using the ANCOM-BC (Analysis of Composition of Microbiomes with Bias Correction) method implemented in QIIME2 (20). Analyses were performed using non-rarefied taxonomic count data. Features with low prevalence across samples were automatically filtered during the ANCOM-BC procedure to improve statistical robustness. Only taxa reaching statistical significance after multiple-testing correction (*q* value threshold) were considered, and non-significant taxa were not included in visualizations. The resulting statistical outputs were visualized using the *ggplot2* package in R.

### Isolation and primary culture of EM cells

Fresh eutopic EM cells were extracted from women without EMS. The EM tissue samples were finely minced and subsequently incubated for 2 h in PBS containing 2.0 mg/mL collagenase type I (Gibco, Waltham, MA, USA) in a humidified incubator maintained at 37°C and 5% CO_2_. Following incubation, the cells were collected by filtering through a 40 μm cell strainer (BD Biosciences, San Jose, CA, USA). The cell culture medium consisted of Dulbecco’s modified Eagle’s medium/F12 (DMEM/F12; Cytiva, Marlborough, MA, USA) supplemented with 10% fetal bovine serum (FBS; Gibco) and 2% penicillin-streptomycin (P/S; Cytiva, Marlborough, MA, USA). The cells were re-suspended in the culture medium and incubated at 37°C and 5% CO_2_. For passaging, when the cells reached 80–90% confluence, 2 mL of 0.25% trypsin-ethylenediamine tetraacetic acid (Gibco) was added to digest the cells for 5 min in the incubator. Then, 1 mL of culture medium was added to stop the digestion. The primary cultured cells from passages 3–5 were used for subsequent experiments.

### *In vitro* co-culture of EM cells with *L. reuteri*: CCK-8 assay

*L. reuteri* (KCTC 3594; NCBI Genome UID: GCA_000010005.1; JGI Genome ID: Gp0131284) and *L. crispatus* (KCTC 5054; NCBI Genome UID: GCA_018987235.1; JGI Genome ID: Gp0130203) were obtained from the Korean Collection for Type Cultures (KCTC). *L. reuteri* was prepared for co-culture with EM cells by cultivation in MRS broth (MB Cell, Seoul, South Korea) at 37°C for 48 h. After cultivation, the bacteria were collected by centrifugation at 3,800 × *g* for 10 min, washed once with PBS, resuspended in PBS, and stored in aliquots at −80°C. The number of colony-forming units (CFU)/mL in the aliquots was determined by plate counting on MRS agar (MB Cell) after thawing. For co-culture with EM cells, aliquots of *Lactobacilli* were thawed and diluted in DMEM/F-12 medium without antibiotics at a multiplicity of infection (MOI) of 1. EM cells cultured in medium without bacteria served as controls. To assess the influence on cell viability, *L. reuteri* were co-cultured with EM cells for up to 24 h.

Cell viability was evaluated using the Cell Counting Kit-8 (CCK-8; Dojindo, Kumamoto, Japan). EM cells were seeded in 96-well plates at a density of 5 × 10^3^ cells per well and incubated for 24 h. Subsequently, 100 μL of DMEM containing 0.5% FBS, with MOI of 1, 5, or 10, or without *L. reuteri*, was added to the wells and incubated for the specified treatment durations. After incubation, 20 μL of CCK-8 solution was added to each well. The plate was covered and incubated in the dark at 37°C for 1–3 h. Optical density (OD) at 450 nm was measured using a VersaMax microplate reader (Molecular Devices, San Jose, CA, USA).

### Addition of estradiol-17-glucuronide to *in vitro* co-culture of EM cells with *L. reuteri*

β-Estradiol 17-(β-d-glucuronide) sodium salt (E2G) was obtained as a solid (Sigma-Aldrich, St. Louis, MO, USA) and dissolved in DMSO to achieve a final concentration of 10 mM. *In vitro* assays were performed in a total reaction volume of 50 μL. Each reaction contained 5 μL of uronate dehydrogenase (1 μM final concentration), 5 μL of the enzyme (final concentration of 30 nM), 10 μL of NAD^+^ (final concentration of 2 mM), and 30 μL of E2G (final concentration of 500 μM), all diluted in assay buffer containing 50 mM HEPES and 50 mM NaCl, with pH varied as required (21).

### Protein isolation and western blot

Proteins were extracted using radioimmunoprecipitation assay buffer (RIPA buffer; Thermo Scientific, Waltham, MA, USA) containing protease inhibitor cocktail (Thermo Scientific). Protein concentrations were measured using the bicinchoninic acid assay kit (Thermo Scientific). Equal amounts of protein (20 μg) were mixed with 5× sodium dodecyl sulfate-polyacrylamide gel electrophoresis (SDS-PAGE) loading buffer (Biosesang, Seongnam, Korea) and heated at 95°C for 5 min. The protein samples were separated via SDS-PAGE on a 10% gel and then transferred onto polyvinylidene fluoride membranes (PVDF; Merck, Darmstadt, Germany). Membranes were blocked with 5% non-fat skim milk in Tris-buffered saline solution (10 mM Tris-HCl [pH 7.4] and 0.5 M NaCl) with Tween-20 (0.1% vol/vol) at 20°C for 1 h. The membranes were incubated with the following specific primary antibodies overnight at 4°C: Phospho-Nrf2 (Ser40) (1:500, Invitrogen, Carlsbad, CA, USA), Nrf2 (E5F1A) (1:250, Cell Signaling Technology, Danvers, MA, USA), Phospho-p44/42 mitogen-activated protein kinases (MAPK) (p-ERK 1/2; 1:500, Cell Signaling Technology), Phospho-p53 (p-p53; 1:1000, Santa Cruz, Dallas, TX, USA), Phospho-nuclear factor (NF)-κB p65 (1:1000, Cell Signaling Technology), Phospho-c-Jun (p-c-jun; 1:1,000, Danvers, MA, USA), Caspase 3 (1:200, Santa Cruz), B-cell lymphoma 2 (Bcl-2) (1:500, Santa Cruz), Bcl-2 Associated X-protein (BAX) (1:500, Santa Cruz), estrogen receptor (ER)-α (1:1,000, Cell Signaling Technology), ER-β (1:1,000, Santa Cruz), Progesterone Receptor (PR; AB-52) (1:200, Santa Cruz), and glyceraldehyde-3-phosphate dehydrogenase (GAPDH) (1:2,000, Santa Cruz). The membranes were then incubated with goat anti-mouse IgG (H + L) or anti-rabbit IgG (H + L) secondary antibody (1:3,000, Thermo Scientific) for 1 h at room temperature. Detection was performed with Super Signal West Pico Plus Chemiluminescent Substrate (Thermo Scientific) solution and imaged in a chemiluminescence imaging system (Image Quant LAS 4000; General Electric, Chicago, IL, USA). The bands were quantified by densitometry using ImageJ software (NIH, Bethesda, MD, USA).

### ELISA-based quantification of β-glucuronidase

Supernatants from the co-culture of EM cells with isolated *L. reuteri* were analyzed for β-glucuronidase concentrations using an ELISA method. Concentrations were measured after 24 h of co-culture using the QuantiChrom β-Glucuronidase Assay Kit (BioAssay Systems, Hayward, CA, USA) following the manufacturer’s instructions. Samples were diluted within the detection range of the assay. To remove EM cells and bacteria, cell culture supernatants were centrifuged at 2,000 × *g* for 5 min after harvesting. The supernatants were then stored at −20°C until measurement. Each experiment was performed in duplicate using EM cells from four different donors. β-Glucuronidase concentrations in the supernatants were determined using a VersaMax microplate reader (Molecular Devices) by comparing the results to standard curves.

### Measurement of estrogen metabolites using LC-MS/MS system: extraction of estrogens and their glucuronide conjugates

Supernatants from the co-culture of EM cells with isolated *L. reuteri* were analyzed for estradiol and E2G concentrations using liquid chromatography with tandem mass spectrometry system (LC-MS/MS system). A 200 µL sample was extracted twice using 500 µL of methyl tert-butyl ether (MTBE) with estrone-^13^C_3_ (2 ng/mL) as an internal standard for non-conjugated estrogen. The organic phase was used to extract estradiol, while the aqueous phase was used for E2G.

The combined organic phases were concentrated to dryness using a speed vacuum concentrator. For dansyl derivatization, 50 µL of dansyl chloride (1 mg/mL in acetone) and 50 µL of 0.1 M sodium bicarbonate buffer (pH 10.4) were added to the dried residue. The mixture was incubated at 60°C for 5 min and then cooled to room temperature. The derivatized sample was diluted 1:1 with methanol and filtered through 0.2 µm nylon filters. The final solution was transferred to inserts placed in LC analysis vials.

To extract the glucuronide, 800 µL of acetonitrile with estradiol-^13^C_3_ (50 ng/mL) as an internal standard for conjugated estrogen was added to the aqueous phase. The mixture was vortexed for 1 min and then centrifuged at 15,294 × *g* for 10 min at 4°C. The supernatant was transferred into LC analysis vials.

### Instrumental analysis utilizing LC-MS/MS system

Estradiol and its glucuronide conjugate were analyzed using an LC-MS/MS system (LCMS-8060, Shimadzu, Kyoto, Japan). A reversed-phase Dikma Navigatorsil C18 analytical column (2.7 µm particle size, 100 × 2.1 mm) was employed for chromatographic separation. The column temperature was maintained at 40°C. The mobile phases were as follows: mobile phase A (5 mM ammonium formate + 5% acetonitrile in water) and mobile phase B (5 mM ammonium formate + 95% acetonitrile in water). The elution gradient program was: 0% B (0–3 min), 0–100% B (3–18 min), 100% B (18–23 min), 100–0% B (23–25 min), and 0% B (25–30 min). The flow rate and injection volume were set to 0.2 mL/min and 5 µL, respectively. Optimal LC-MS/MS parameters were as follows: nebulizing gas flow, 3 L/min; drying gas flow, 10 L/min; and heating gas flow, 10 L/min. The MS system was operated with the following temperatures: DL temperature, 250°C; interface temperature, 350°C; and heat block temperature, 400°C. Details of the multiple reaction monitoring (MRM) transitions are presented in the Table S1.

### Statistical analysis

Microbiome analysis using NGS involved statistical comparisons of species diversity (diversity indices) and microbial community composition (relative abundance) across different sites (vagina, endometrium, and PF) in both the EMS and control groups. The Kruskal-Wallis rank-sum test (22) was used for comparisons among three groups, and the Wilcoxon rank-sum test (23, 24) was employed for comparisons between the two groups. To provide an estimate of effect size, log_2_ fold change (log_2_FC) values were calculated based on the OTU-level relative abundance data. Log_2_FC was defined as log_2_FC = log_2_(EMS + epsilon) − log_2_(Control + epsilon), where epsilon represents a small pseudocount added to avoid undefined values due to zero abundance. The pseudocount was set to 1 × 10^−6^, which is smaller than the minimum non-zero value observed in the data set. Additionally, to analyze microbial community composition (relative abundance) across the groups, a linear discriminant effect size (LEfSe) analysis (25) was performed to identify microorganisms that exhibited significant differences between the comparison groups, with the magnitude of these differences expressed through the LDA score.

The results of the *in vitro* experiments are presented as the mean ± standard deviation (SD). The data were checked to determine whether they met the requirements for a normal distribution using the Shapiro-Wilk test. Continuous variables were compared using the Student’s *t*-test or Mann-Whitney *U*-test where appropriate. SPSS v.27.0 (IBM, Armonk, NY, USA) and GraphPad Prism program (GraphPad Software Inc, San Diego, CA, USA) were used for statistical analyses. All statistical analyses were visualized using GraphPad Prism program. A *P* value < 0.05 was considered statistically significant.

## RESULTS

### NGS-based profiling of the vaginal, EM, and peritoneal microbiome in EMS

**Fig 1:**
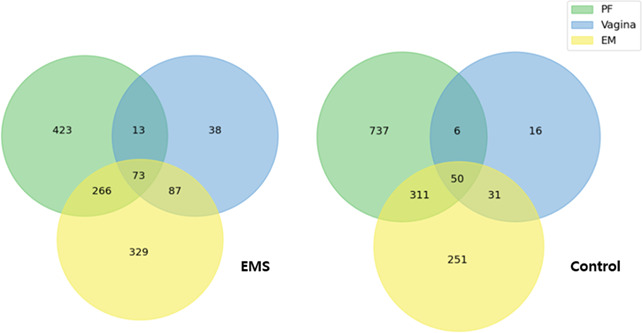
Number of microbiome species identified in the vagina, endometrium (EM), and peritoneal fluid (PF) of women with endometriosis (EMS) and controls.

A total of 45 patients were enrolled, and 41 were included after quality filtering. Baseline characteristics of participants are presented in Table 1. In total, 239 microbiome species were identified in the vagina, 1,077 in the endometrium, and 1,433 in the PF (Fig. 1). Between EMS and control groups, 75 vaginal, 321 EM, and 446 PF species were shared. The mean sequence counts were 43,396 (vagina), 44,265 (EM), and 30,295 (PF). Overall, microbial abundance was greatest in the vagina and endometrium, whereas microbial diversity increased progressively from the vagina to the endometrium and PF. Species-level differences in the microbiome between women with EMS and controls across the vagina, endometrium, and PF are summarized in Table 2.

**TABLE 1:** Baseline characteristics of the study population^*a*^^,^^*b*^

	Endometriosis group (*n* = 27)	Control group (*n* = 14)	*P* value
Age, years	32.4 ± 4.4	31.7 ± 4.6	0.413
Height, cm	161.7 ± 4.5	160.8 ± 4.2	0.479
Weight, kg	54.1 ± 7.2	53.1 ± 6.2	0.321
Size of endometrioma, cm	5.1 ± 3.1		
Revised ASRM classification, *N* (%)			
I, II	1 (3.70)		
III	16 (59.26)		
IV	10 (37.04)		
*Revised ASRM classification total score	29 (25–108)		

^*a*^ The values are expressed as the mean ± standard deviation.

^*b*^ ASRM, American Society for Reproductive Medicine.

**TABLE 2:** Species-level differences in microbiome composition between women with endometriosis (EMS) and controls identified by the Wilcoxon rank-sum test with corresponding log_2_ fold change (log_2_FC)^*a*^

Site	Species	Enriched group	log_2_FC EMS/control	*P* value
Vagina	*Dialister micraerophilus*	EMS	6.280005911	0.0089
	*Porphyromonas bennonis*	EMS	6.61608256	0.034
	*Lactobacillus iners*	Control	−1.268865256	0.015
	*Lactobacillus reuteri*	Control	−1.609898408	0.0085
Endometrium	*Acidibrevibacterium fodinaquatile*	Control	−6.995873477	0.047
	*Actinomadura rifamycini*	Control	−6.830759741	0.047
	*Aeromicrobium panaciterrae*	Control	−8.246618483	0.047
	*Afifella pfennigii*	Control	−8.46598842	0.047
	*Altererythrobacter rigui*	Control	−5.327575631	0.047
	*Arenimonas subflava*	Control	−7.183327958	0.047
	*Campylobacter ureolyticus*	EMS	10.70206056	0.041
	*Chryseobacterium greenlandense*	Control	−8.134212895	0.0033
	*Corynebacterium tuberculostearicum*	EMS	4.78902972	0.02
	*Desulfonatronum alkalitolerans*	Control	−7.346036343	0.047
	*Desulfovibrio piger*	Control	−6.574749807	0.047
	*Dongia mobilis*	Control	−8.554760688	0.047
	*Edaphobacter dinghuensis*	Control	−4.773795323	0.047
	*Howardella ureilytica*	EMS	5.355473853	0.026
	*Insolitispirillum peregrinum*	Control	−6.620247319	0.047
	*Lactobacillus reuteri*	Control	−0.540402898	0.0061
	*Lysobacter dokdonensis*	Control	−6.157156594	0.047
	*Lysobacter niabensis*	Control	−6.407144959	0.047
	*Mycobacterium marinum*	Control	−5.969488254	0.047
	*Natranaerovirga pectinivora*	Control	−5.232825367	0.047
	*Nocardioides alpinus*	Control	−7.257245755	0.047
	*Pelobacter carbinolicus*	Control	−5.337971042	0.047
	*Povalibacter uvarum*	Control	−6.798125238	0.047
	*Prevotella timonensis*	EMS	6.799906456	0.018
	*Pseudoduganella violaceinigra*	Control	−7.489881158	0.047
	*Pseudoflavonifractor capillosus*	Control	−10.11202371	0.047
	*Pseudoxanthomonas wuyuanensis*	Control	−7.55361836	0.047
	*Rhodanobacter glycinis*	Control	−8.086466305	0.013
	*Smithella propionica*	Control	−8.229049939	0.047
	*Sphingobium mellinum*	Control	−7.384932352	0.047
	*Streptomyces aomiensis*	Control	−3.495528313	0.047
	*Streptomyces chlorus*	Control	−8.161137516	0.047
Peritoneal fluid	*Thauera chlorobenzoica*	EMS	8.052636498	0.026

^*a*^ log_2_FC, log_2_ fold change of relative abundance.

Vaginal microbiome composition of women with EMS compared to controls across taxonomic levels is illustrated in Fig. S1. The most abundant phyla were Firmicutes, Actinobacteria, Bacteroidetes, Proteobacteria, and Tenericutes. At the genus level, *Lactobacillus*, *Streptococcus*, *Gardnerella*, *Atopobium*, and *Prevotella* predominated, while at the species level, *Lactobacillus crispatus (L. crispatus)*, *Lactobacillus iners (L. iners)*, *Streptococcus agalactiae*, *Gardnerella vaginalis*, *Atopobium vaginae*, and *Lactobacillus gasseri* (*L. gasseri*) were most abundant. Diversity analysis showed no significant differences between EMS and controls, as assessed by α-diversity indices (Shannon, Inverse Simpson, and Chao1) and β-diversity measures (UPGMA and PCoA). Nevertheless, the Wilcoxon rank-sum test identified significant differences in specific taxa, with *Sutterella* enriched in EMS at the genus level and four species—*Dialister micraerophilus*, *Porphyromonas bennonis*, *L. iners*, and *L. reuteri*—differing between groups.

The EM microbiome of women with EMS compared with controls is presented in Fig. S2. The dominant phyla were Firmicutes*,* Actinobacteria, and Bacteroidetes. At the genus level, *Lactobacillus*, *Gardnerella*, and *Streptococcus* were predominant, while the leading species included *L. iners*, *L. crispatus*, *G. vaginalis*, *S. agalactiae*, and *L. gasseri*. Analyses of α- and β-diversity revealed no significant differences between the two groups. Abundance testing identified 32 species with differential representation: four species*—Campylobacter ureolyticus*, *Corynebacterium tuberculostearicum*, *Howardella ureilytica*, and *Prevotella timonensis—*were enriched in women with EMS, whereas 28 species, including *Chryseobacterium greenlandense*, *L. reuteri*, and *Rhodanobacter glycinis*, were more abundant in controls.

Figure S3 illustrates the peritoneal microbiome composition. The most abundant phyla were Proteobacteria, Firmicutes, and Bacteroidetes, while the predominant genera included *Photobacterium*, *Bacteroides*, and *Lactobacillus*. The leading species were *Photobacterium piscicola*, *Bacteroides vulgatus*, *Lactobacillus aviarius*, *Pediococcus pentosaceus*, and *Aerosakkonema funiforme*. Analyses of α- and β-diversity revealed no significant differences between the EMS and control groups. Differential abundance testing identified Thauera chlorobenzoica as enriched in EMS.

To further confirm that the observed species-level patterns were not dependent on OTU clustering, we additionally performed an ASV-based analysis. The ASV profiles demonstrated overall concordant compositional patterns across compartments and study groups, supporting the robustness of the species-level signals identified in the OTU-based analysis (Fig. S4).

### Validation of differentially abundant taxa using LEfSe and ANCOM-BC

Differential abundance analyses of vaginal, EM, and PF microbiomes were performed using both LEfSe and the compositionality-aware method ANCOM-BC. In the vaginal microbiome, LEfSe identified several discriminant taxa, with members of *Bifidobacteriaceae*, *Sutterella*, *P. bennonis*, and *D. micraerophilus* enriched in EMS, whereas *L. iners* and *L. reuteri* were enriched in controls (Fig. S1). ANCOM-BC confirmed a more conservative subset, including enrichment of *D. micraerophilus*, *Prevotella disiens*, and *Hoylesella timonensis* in EMS and *L. iners* and *L. reuteri* in controls (Fig. S5).

In the EM microbiome, LEfSe likewise identified multiple taxa differentiating groups, including enrichment of *C. ureolyticus*, *C. tuberculostearicum*, *H. ureilytica*, and *D. micraerophilus* in EMS and several taxa enriched in controls (Fig. S2). ANCOM-BC again yielded a more restricted set of significant taxa but confirmed enrichment of key EMS-associated species, such as *H. timonensis*, *P. disiens*, *C. tuberculostearicum*, and *D. micraerophilus*, while *L. iners* remained enriched in controls (Fig. S6).

In PF, LEfSe detected only a limited number of EMS-enriched taxa (Fig. S3), whereas ANCOM-BC identified a broader set of differentially abundant taxa (Fig. S7). Despite differences in sensitivity between methods, the direction of enrichment was consistent across analyses for all anatomical sites, supporting the robustness of the observed microbial differences.

### *In vitro* effects of *L. reuteri* on EM cells: determination of optimal co-culture conditions

**Fig 2:**
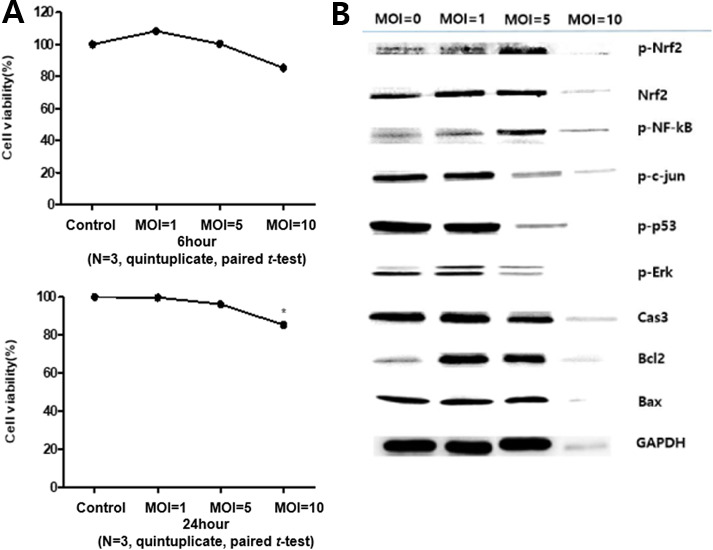
Optimization of co-culture conditions for *L. reuteri*. (**A**) Cell viability of EM cells co-cultured with *L. reuteri* at multiplicities of infection (MOIs) of 1, 5, and 10, assessed at 6 h (top) and 24 h (bottom) using the CCK-8 assay. (**B**) Protein expression of EM cells following 24 h co-culture with *L. reuteri* at MOI 1, 5, and 10, evaluated by western blot analysis. Data are presented as mean ± SEM (*N* = 3 independent experiments, quintuplicate). *P* < 0.05 was considered statistically significant.

Cell viability of EM cells co-cultured with *L. reuteri* at different multiplicities of infection (MOI = 1, 5, and 10) was assessed using a CCK-8 assay at 6 and 24 h (Fig. 2A). At 6 h, cell viability did not differ significantly across MOIs, although a modest decrease was observed at MOI = 10. In contrast, after 24 h, viability declined in a dose-dependent manner, with a significant reduction observed at MOI = 10.

To further investigate the impact of *L. reuteri* on EM cells, western blot analysis was performed after 24 h of co-culture to assess the expression of key proteins associated with endometriosis (Fig. 2B). Consistent with the CCK-8 results, protein expression progressively decreased with increasing MOI, and the reduction was particularly pronounced at MOI values above 1.

### Modulation of EMS-related gene expression by *L. reuteri* and E2G in EM cells

**Fig 3:**
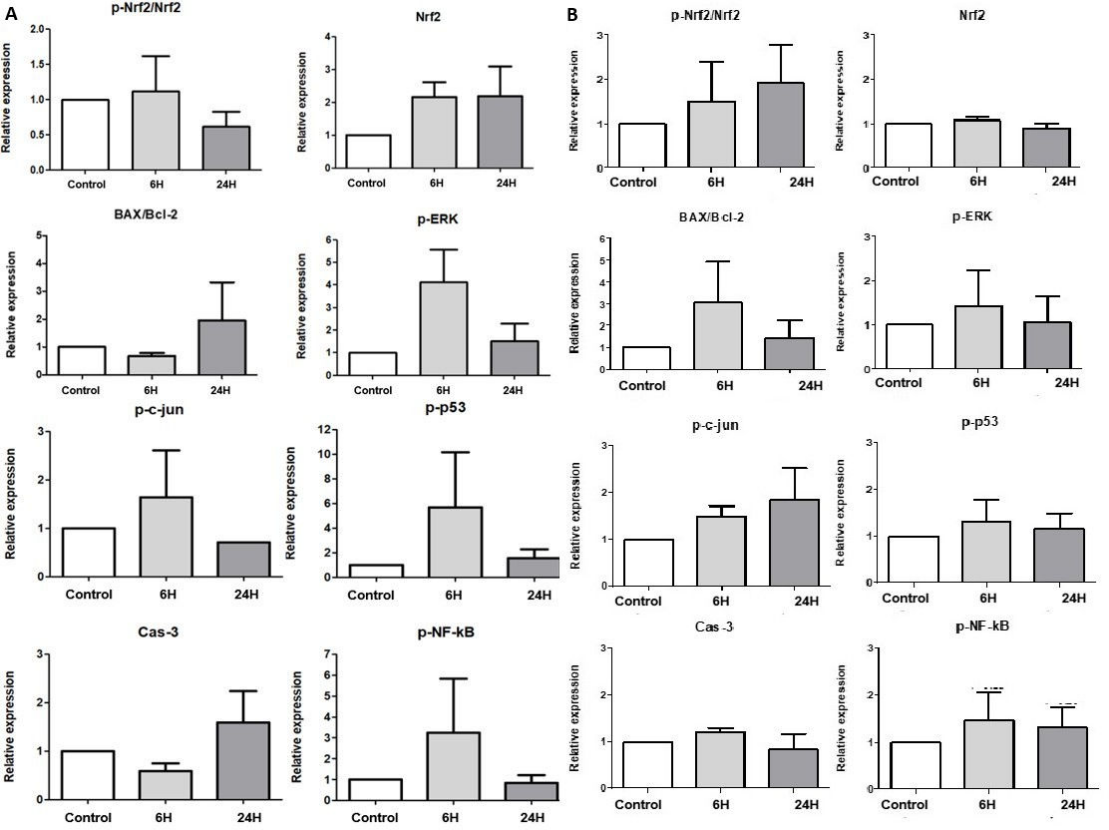
Effects of *Lactobacillus* co-culture on protein expression in EM cells. (**A**) Protein expression of endometriosis-associated markers in EM cells after 6 and 24 h co-culture with *L. reuteri* at MOI = 1. No significant alterations were observed. Data are presented as mean ± SEM (*n* = 5). (**B**) Protein expression of endometriosis-associated markers in EM cells after 6 and 24 h co-culture with *L. crispatus* under baseline conditions. No significant alterations were observed. Data are presented as mean ± SEM (*n* = 3). Statistical significance was defined as *P* < 0.05.

As a comparative microbial control, EM cells were co-cultured with *L. crispatus* in parallel with *L. reuteri*. After 6 and 24 h of co-culture, the expression levels of EMS-associated proteins showed no statistically significant changes in cells exposed to either species. Although moderate variations were observed—such as slight increases in Nrf2, BAX/Bcl-2, p-p53, p-ERK, and Cas-3, along with decreases in the p-Nrf2/Nrf2 ratio, p-c-Jun, and p-NF-κB—none reached statistical significance under baseline conditions (Fig. 3). These findings indicate that bacterial exposure alone does not alter cellular signaling in the absence of hormonal stimulation.

**Fig 4:**
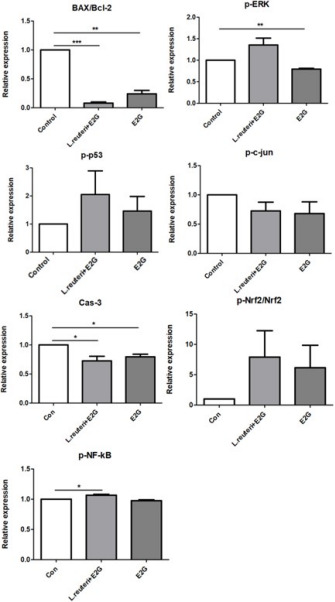
Effects of *Lactobacillus reuteri* and estradiol-17-glucuronide (E2G) on signaling and apoptosis-related proteins in EM cells. Relative protein expression levels of BAX/Bcl-2, p-ERK, p-p53, p-c-Jun, Cas-3, p-Nrf2/Nrf2, and p-NF-κB in EM cells cultured for 24 h under the following conditions: control, *L. reuteri* alone (MOI = 1), E2G alone (10 mM), and *L. reuteri* + E2G. Data are presented as mean ± SEM (*n* = 5). Statistical comparisons were performed relative to control unless otherwise indicated. *P*(*) < 0.05; *P*(**) < 0.01; and *P*(***) < 0.001.

When E2G was added to simulate follicular phase conditions, treatment with E2G alone induced significant changes in selected apoptosis-related markers, including reductions in the BAX/Bcl-2 ratio and Cas-3 levels compared with control (Fig. 4). Under these estrogenic conditions, co-culture with *L. reuteri* produced additional alterations in specific signaling pathways, including an increase in p-NF-κB expression at MOI = 1 (Fig. 4). Direct comparisons between the E2G-only and *L. reuteri* + E2G groups indicated that these responses were not uniformly additive across all endpoints, suggesting condition-dependent modulation rather than consistent synergistic effects.

When EM cells were co-cultured with *L. reuteri* at a lower bacterial load (MOI = 0.5) in the presence of E2G, a broadly comparable but not identical pattern was observed. Notably, p-NF-κB expression did not increase at this MOI and instead showed a decreasing trend, indicating that this response was dependent on bacterial density. Nevertheless, significant alterations were detected in other markers, including the p-Nrf2/Nrf2 ratio, BAX/Bcl-2 ratio, and p-p53 levels, which shifted toward profiles consistent with enhanced cell survival signaling (Fig. S8). Together, these findings indicate that host cellular responses to *L. reuteri* under estrogenic conditions vary according to microbial load rather than following a single directional pattern.

### Effects of *L. reuteri* and E2G on estrogen pathways in EM cells

Because estrogen exerts its biological actions through receptor-mediated signaling as well as metabolic conversion, we examined both receptor expression and estrogen metabolites in EM cells co-cultured with *L. reuteri* and E2G.

#### Receptor expression

**Fig 5:**
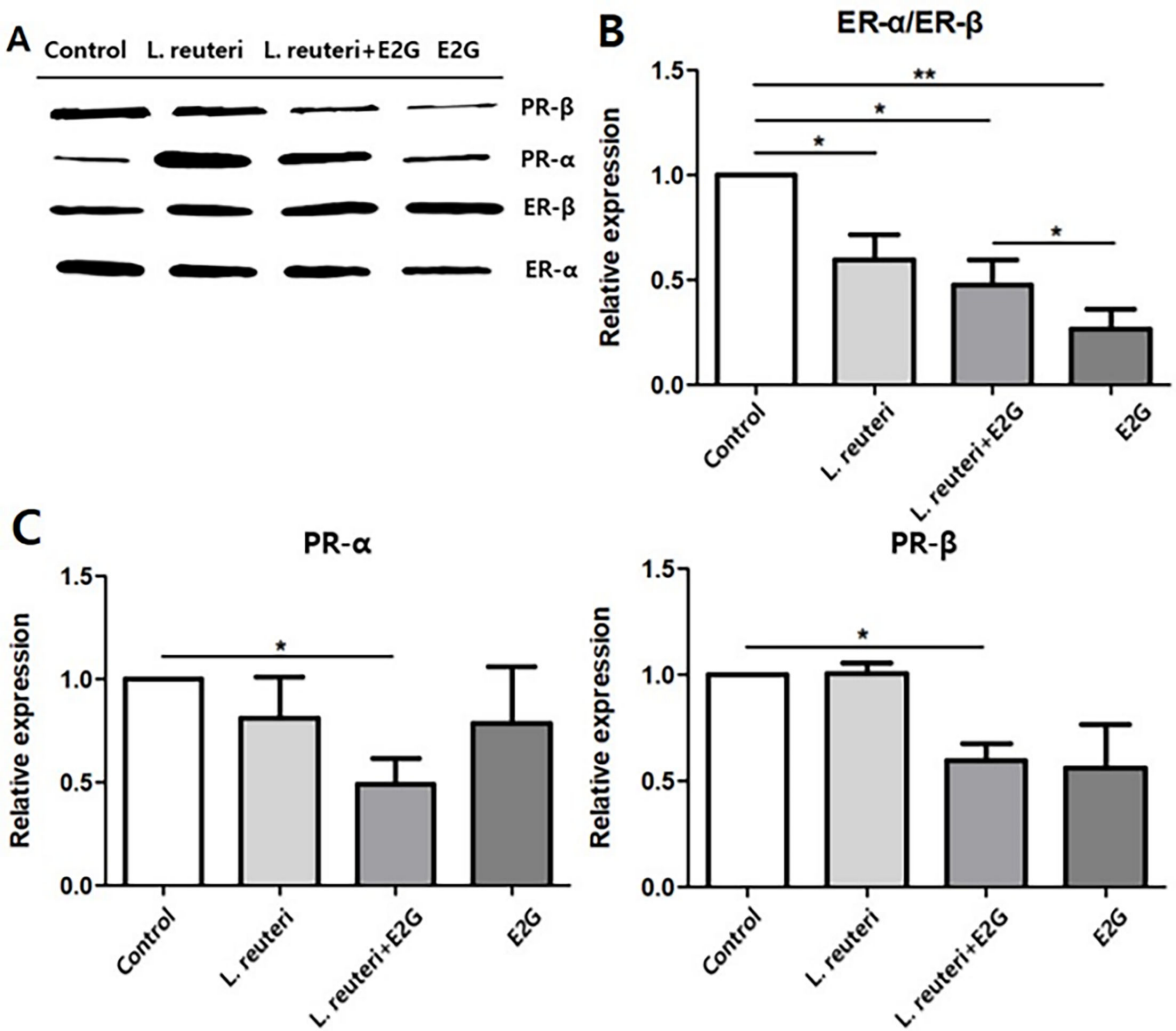
Expression of estrogen and progesterone receptors (ER and PR) in EM cells co-cultured with *L. reuteri* and estradiol-17-glucuronide (E2G). (**A**) Representative western blot images showing ER-α, ER-β, PR-α, and PR-β protein expression in cells cultured under the following conditions: control, *L. reuteri* alone, *L. reuteri* + E2G (10 mM), and E2G alone. (**B**) Quantification of the ER-α/ER-β ratio. A significant reduction was observed in all treatment groups compared with control, with the greatest decrease detected in the E2G-only condition. (**C**) Relative expression of PR-α and PR-β. Both isoforms exhibited downward trends under treatment conditions; however, statistically significant reductions were observed only in the *L. reuteri* + E2G group compared with control. Data are presented as mean ± SEM. (*N* = 5, MOI = 1, E2G: 10 mM). *P*(*) < 0.05; *P*(**) < 0.01.

When EM cells were co-cultured with *L.* reuteri and/or E2G for 24 h (Fig. 5), the ER-α/ER-β ratio decreased across treatment conditions, with the greatest reduction observed in the E2G-only group. In contrast, PR-α and PR-β expressions were significantly reduced only in the *L. reuteri* + E2G co-culture group, whereas no statistically significant changes were observed in the *L. reuteri*-only or E2G-only groups. These findings suggest that *L. reuteri* may modulate progesterone receptor (PR) expression under estrogenic conditions.

#### Estrogen metabolism

**Fig 6:**
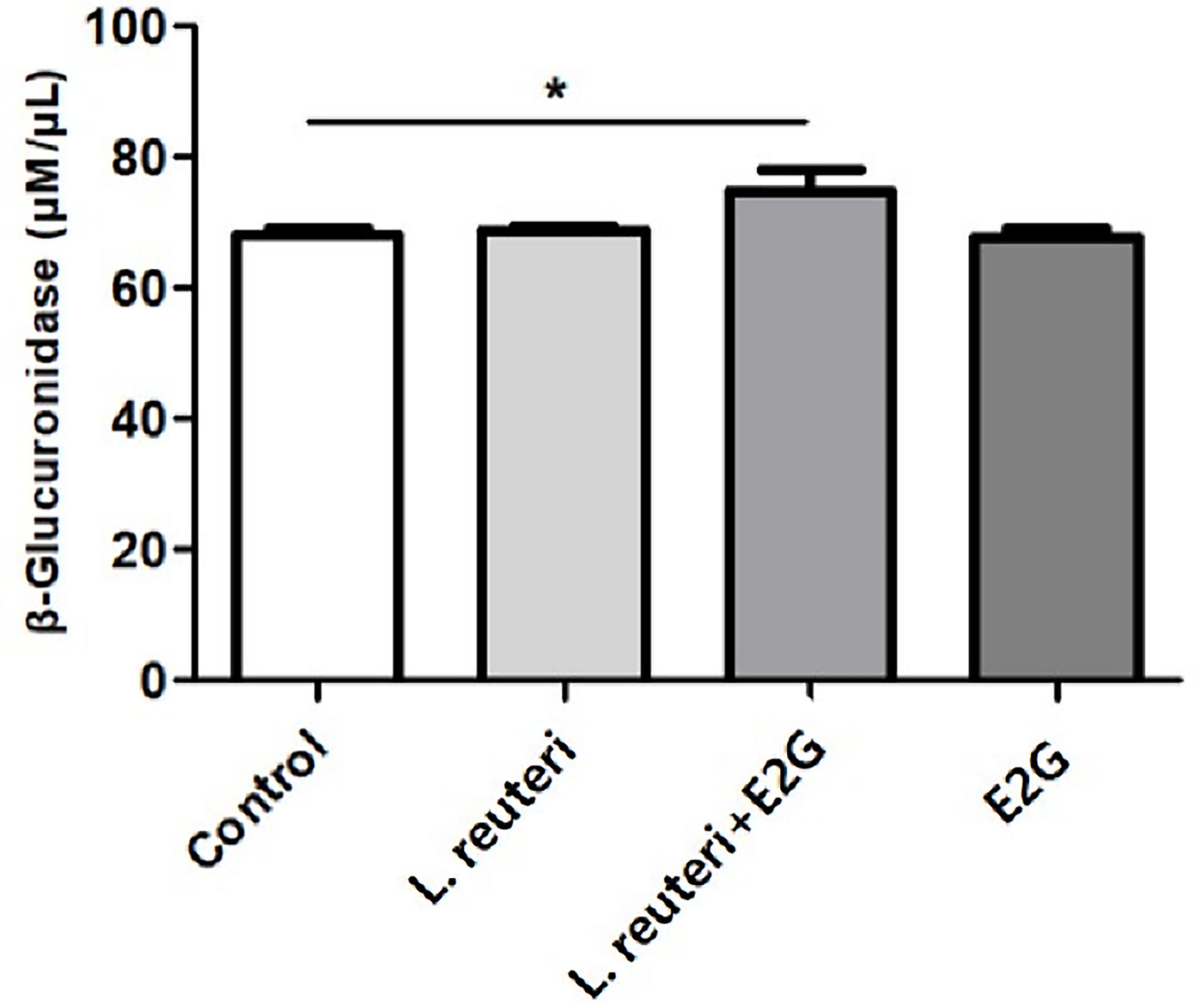
β-glucuronidase production in EM cells co-cultured with *L. reuteri* and estradiol-17-glucuronide (E2G). β-glucuronidase levels in the culture medium were measured after 24 h under four conditions: control, *L. reuteri* alone, *L. reuteri* + E2G (10 mM), and E2G alone. A significant increase in β-glucuronidase production was observed only in the *L. reuteri* + E2G group compared with the control. Data are presented as mean ± SEM (*N* = 5, MOI = 1, E2G = 10 mM). *P < 0.05.

To evaluate estrogen metabolism, β-glucuronidase activity and estradiol production were measured after 24 h of co-culture (Fig. 6; Table 3). β-glucuronidase levels were significantly elevated only under the *L. reuteri* + E2G condition compared with the control. However, despite this increase, the estradiol/E2G ratio showed no evidence of enhanced conversion of E2G to estradiol in the co-culture.

**TABLE 3:** Estradiol-17-glucuronide (E2G) and estradiol levels measured by LC-MS/MS after 24 h co-culture^*a*^^,^^*b*^

	E2G	Estradiol	Estradiol/E2G
Control			
*L. reuteri*			
*L. reuteri +* E2G	0.36 (0.02)	3.32 (2.21)	9.11 (5.75)
E2G	0.35 (0.08)	2.97 (2.06)	8.68 (5.63)
*P* value	0.009	0.656	0.837

^*a*^ Measurements were performed in four groups: EM cells alone (control), EM cells co-cultured with *L. reuteri* (MOI = 1), EM cells co-cultured with *L. reuteri* and E2G (10 mM), and EM cells cultured with E2G alone.

^*b*^ E2G, Estradiol-17-glucuronide; LC-MS/MS, liquid chromatography–tandem mass spectrometry; *L. reuteri*,* Lactobacillus reuteri*; MOI, multiplicity of infection.

## DISCUSSION

In this study, microbiome composition in samples obtained from the vagina, endometrium, and PF was compared between women with EMS and control women using 16S rRNA gene sequencing. Although no overall differences in α or β diversity were observed between the two groups, specific bacterial taxa demonstrating significant differences were identified at each anatomical site. Among these, *L. reuteri* was the only species that exhibited significant differences in both the vagina and endometrium, with a relatively greater disparity between the two groups compared to other species.

To evaluate the potential role of *L. reuteri* in EMS, EM cells were co-cultured with the bacterium for 24 h. At MOI = 1 under baseline conditions, no significant changes in EMS-related protein expression were observed, indicating minimal direct effects in the absence of hormonal stimulation. However, when estrogenic conditions were simulated by E2G supplementation, co-culture with *L. reuteri* altered selected signaling pathways, including a reduced BAX/Bcl-2 ratio and increased p-NF-κB expression at MOI = 1. Notably, this response was not uniform across bacterial loads, as the p-NF-κB increase was not observed at MOI = 0.5, indicating that host cellular responses to *L. reuteri* are dependent on microbial density and hormonal context rather than representing a consistent directional effect.

To clarify how *L. reuteri* may interact with estrogenic signaling, hormone receptor expression and estrogen metabolism were further examined. While estrogen receptor levels were not significantly affected, PR-α and PR-β expression were markedly reduced in the presence of *L. reuteri* and E2G. In addition, consistent with its reported enzymatic activity, co-culture with *L. reuteri* led to a significant increase in β-glucuronidase secretion. Nevertheless, quantitative LC-MS/MS analysis revealed no change in estradiol levels or in the estradiol/E2G ratio, indicating that under the experimental conditions used in this study, the conversion of E2G to estradiol was not facilitated.

These *in vitro* findings appear paradoxical when compared with microbiome profiling results. Both the NGS data from this study and previous reports have consistently shown that women with EMS exhibit reduced levels of *Lactobacillus* species, including *L. reuteri*, compared with controls (26). Similar reductions have also been noted in infertility and other gynecological disorders, supporting the view that *Lactobacillus* generally contributes to reproductive tract health (27, 28).

However, our functional data indicate that the biological effects of *L. reuteri* are context-dependent rather than uniformly protective. Under estrogenic conditions, co-culture with *L. reuteri* altered selected signaling pathways associated with cell survival, including a reduced BAX/Bcl-2 ratio at both MOIs and increased p-NF-κB expression at MOI = 1, whereas this inflammatory signal was not observed at MOI = 0.5. These findings suggest that host cellular responses to *L. reuteri* vary according to microbial load and hormonal milieu rather than reflecting a consistent directional effect. The transient nature of these molecular changes further supports the possibility of an acute, non-sustained response.

One explanation is that the presence of a single bacterial species may be insufficient to reproduce the regulatory dynamics of the native EM microbiome. Unlike the vaginal microbiota, which is typically dominated by *Lactobacillus*, the EM microbial community is more diverse, with *Lactobacillus* comprising a substantially smaller proportion (29). Such diversity may buffer or modulate the effects of individual species, thereby limiting excessive pro-survival or inflammatory signaling.

Taken together, these findings suggest that *L. reuteri*, although traditionally regarded as a beneficial commensal, may exert variable biological effects depending on microbial density and host–microbe interactions. Notably, the observed reduction in PR expression and increase in β-glucuronidase activity—without evidence of enhanced estrogen reactivation—indicate that its influence may involve selective modulation of steroid-related signaling rather than global hormonal activation. The relatively lower abundance of *Lactobacillus* in the endometrium compared with the vagina may therefore reflect a physiological equilibrium that constrains prolonged signaling activation and helps maintain tissue homeostasis.

Because certain bacterial strains, including *L. reuteri*, have been reported to secrete β-glucuronidase, these species may contribute to the estrobolome and influence local estrogen metabolism. Estradiol produced by the ovaries is normally metabolized in the liver into conjugated estrogens of lower potency; however, β-glucuronidase secreted by various bacteria can deconjugate these metabolites back into active estradiol, creating an estrogenic environment that may promote the development of estrogen-dependent diseases such as EMS (7, 30).

Under physiological conditions, *Lactobacillus* in the female reproductive tract typically does not come into direct contact with blood. During menstruation, however, shedding of the EM lining exposes blood vessels, providing an opportunity for *Lactobacillus* to interact with conjugated estrogens present in menstrual blood. To investigate these dynamics—particularly the interaction between estrogen metabolites and the microbiome—we established an experimental co-culture model of EM cells and *L. reuteri*, supplemented with E2G, the most common conjugated estrogen. This system was designed to simulate menstrual conditions and assess potential microbial effects on estrogen metabolism.

As a comparative microbial control, we also co-cultured EM cells with *L. crispatus* under baseline conditions (Fig. 3B). *L. crispatus* is widely regarded as a beneficial commensal species and, unlike *L. reuteri*, is not commonly associated with β-glucuronidase activity. Consistent with this distinction, co-culture with *L. crispatus* did not alter apoptosis- or signaling-related protein expression in the absence of hormonal supplementation. Importantly, this indicates that the observed cellular responses are unlikely to be attributable merely to nonspecific effects of bacterial co-culture itself. Rather, these findings support our rationale for focusing mechanistic investigations on *L. reuteri*, a species with greater theoretical potential to interact with estrogen metabolites through enzymatic activity.

The observed alterations in BAX/Bcl-2 and p-NF-κB expression indicate that interactions between *L. reuteri* and E2G can influence cellular signaling in EM cells; however, these effects were not uniform across experimental conditions and varied according to bacterial density. At MOI = 1, co-culture with *L. reuteri* in the presence of E2G was associated with shifts consistent with enhanced survival signaling, whereas at MOI = 0.5 the pattern differed, indicating that the direction and magnitude of host responses are context-dependent rather than consistently synergistic. These findings prompted further examination of estrogen-related pathways potentially modulated by *L. reuteri*. Although the ER-α/ER-β ratio remained unchanged, PR-α and -β expression was significantly reduced only under *L. reuteri* + E2G conditions, suggesting that microbial–hormonal interactions may contribute to progesterone resistance, a hallmark feature of EMS.

Although this study did not demonstrate a direct role of *L. reuteri* in the metabolism or signaling of female sex steroids, the observed alterations in PR expression and β-glucuronidase activity suggest potential indirect interactions with hormone-related pathways. Notably, β-glucuronidase—a key enzyme involved in estrogen recycling—was significantly elevated only under the *L. reuteri* + E2G condition, whereas estradiol concentrations and the estradiol/E2G ratio remained unchanged compared with E2G-treated cells. These findings indicate that the conversion of E2G to estradiol was not facilitated under the current *in vitro* conditions, possibly due to environmental limitations such as suboptimal temperature for deconjugation or the relatively low enzymatic activity of *L. reuteri*.

Recently, Wei et al*.* reported elevated β-glucuronidase expression in bowel and uterosacral ligament lesions from women with EMS compared with normal endometrium (10). Their study further demonstrated that β-glucuronidase contributes to EMS progression by inducing macrophage dysfunction both *in vitro* and *in vivo*. Although enhanced conversion of E2G to estradiol was not observed in our co-culture experiments, the findings of Wei et al*.* suggest that β-glucuronidase may nonetheless exert direct effects on intracellular signaling pathways, thereby contributing to the pathophysiology of EMS.

It is important to acknowledge that, while these results provide insights into the molecular interactions among EM cells, *L. reuteri*, and estrogen metabolites, the experimental design cannot fully recapitulate the complex environment of menstrual blood, which harbors diverse microbial communities. Our findings highlight the importance of considering both hormonal and microbial influences in EMS and indicate that microbial effects may be hormone-dependent and pathway-specific rather than uniformly synergistic, particularly given that E2G alone accounted for a substantial proportion of the observed changes in apoptosis-related markers. In particular, these results suggest that even well-recognized probiotics, when applied as single-strain formulations, may exert unintended adverse effects on the female reproductive tract under estrogenic conditions.

In addition, because our analysis targeted the V3–V4 region of the 16S rRNA gene, species-level discrimination within closely related taxa such as *Lactobacillus* may be limited; future studies employing long-read sequencing or additional hypervariable regions could improve taxonomic resolution. Although *L. crispatus* was included as an additional microbial control under baseline conditions, estrogen-supplemented co-culture experiments with this species were not performed, and comparative analyses across multiple *Lactobacillus* species under hormonally defined conditions will be important for clarifying species-specific microbial effects. Future studies should therefore investigate the interactions of multiple bacterial species across varying hormonal milieus to more accurately reflect *in vivo* conditions. Such efforts will be essential to validate these observations *in vivo* and in clinical settings and may ultimately inform novel therapeutic strategies for EMS that harness the protective potential of beneficial microbiota within the reproductive tract.

### Conclusion

This study characterized microbiome composition across reproductive tract compartments and examined the functional effects of *L. reuteri* in EMS. Microbiome profiling revealed a reduced abundance of *L. reuteri* in women with EMS, suggesting a potentially protective association. In contrast, *in vitro* experiments under estrogenic conditions demonstrated context-dependent pro-survival and anti-apoptotic cellular responses, accompanied by reduced PR expression and increased β-glucuronidase activity. Collectively, these findings indicate that microbial influences in EMS are conditional rather than uniformly beneficial and highlight the need for *in vivo* studies to define how commensal bacteria modulate disease-relevant signaling pathways and therapeutic targets.

## Data Availability

The 16S rRNA gene sequencing raw data for our samples have been deposited in the NCBI Sequence Read Archive (SRA) under accession number PRJNA1431678.

## SUPPLEMENTAL MATERIAL

Fig. S1 spectrum.03689-25-s0001.TIF Vaginal microbiome composition in women with endometriosis compared to controls.

Fig. S2 spectrum.03689-25-s0002.tif Endometrial microbiome composition in women with endometriosis and controls.

Fig. S3 spectrum.03689-25-s0003.tif Composition and differential analysis of the peritoneal microbiome in women with endometriosis and controls.

Fig. S4 spectrum.03689-25-s0004.tif ASV-based taxonomic composition of the genital tract microbiome at genus and species levels.

Fig. S5 spectrum.03689-25-s0005.tif ANCOM-BC-based differential abundance analysis of the vaginal microbiome in endometriosis and controls.

Fig. S6 spectrum.03689-25-s0006.tif ANCOM-BC-based differential abundance analysis of the endometrial microbiome in women with endometriosis and control subjects.

Fig. S7 spectrum.03689-25-s0007.tif ANCOM-BC-based differential abundance analysis of the peritoneal microbiome in endometriosis and control groups.

Fig. S8 spectrum.03689-25-s0008.tif Modulation of gene expression in endometrial cells under simulated follicular phase conditions (E2G + *L. reuteri*).

Supplemental material spectrum.03689-25-s0009.docx Table S1; Supplemental figure legends.

ASM does not own the copyrights to Supplemental Material that may be linked to, or accessed through, an article. The authors have granted ASM a non-exclusive, world-wide license to publish the Supplemental Material files. Please contact the corresponding author directly for reuse.
