# Supplementary figures and images for: Microbiome in women with endometriosis and the in vitro effects of Lactobacillus reuteri on human endometrium
Source: Microbiol Spectr. 2026 May 6;14(6):e03689-25. doi: 10.1128/spectrum.03689-25 (PMC13228028; doi:10.1128/spectrum.03689-25)

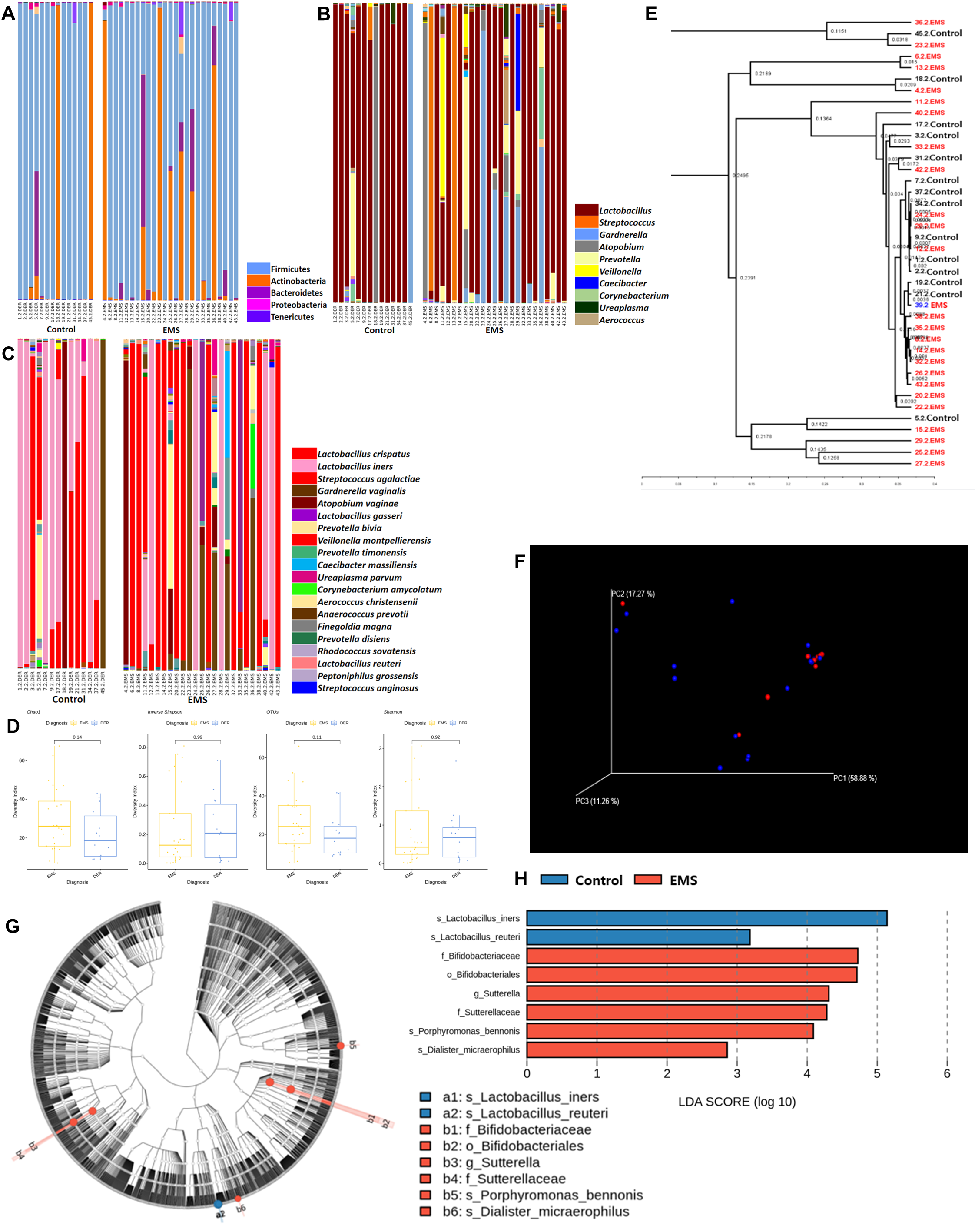

Supplement: Fig. S1 — Vaginal microbiome composition in women with endometriosis compared to controls. [file spectrum.03689-25-s0001.tif]

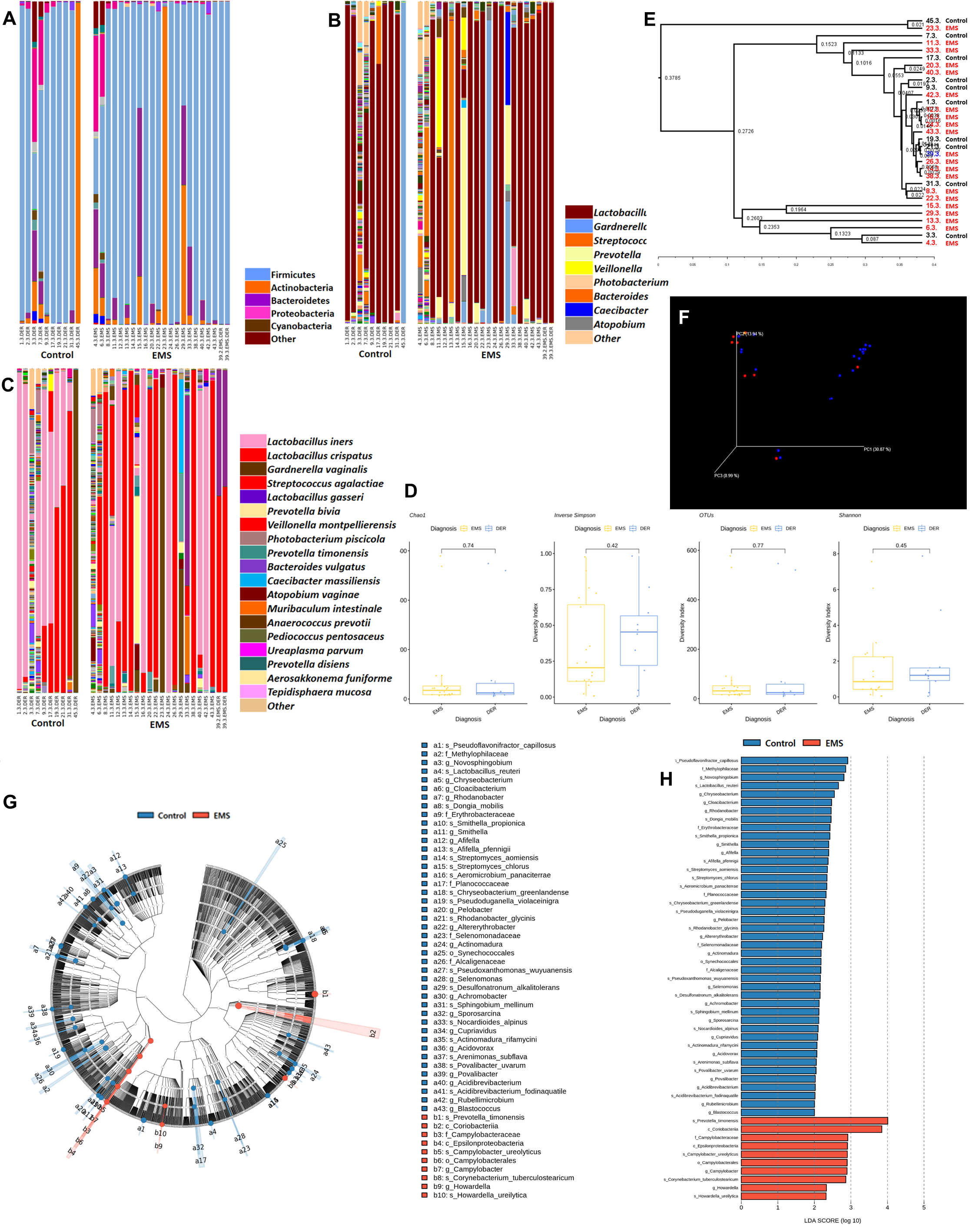

Supplement: Fig. S2 — Endometrial microbiome composition in women with endometriosis and controls. [file spectrum.03689-25-s0002.tif]

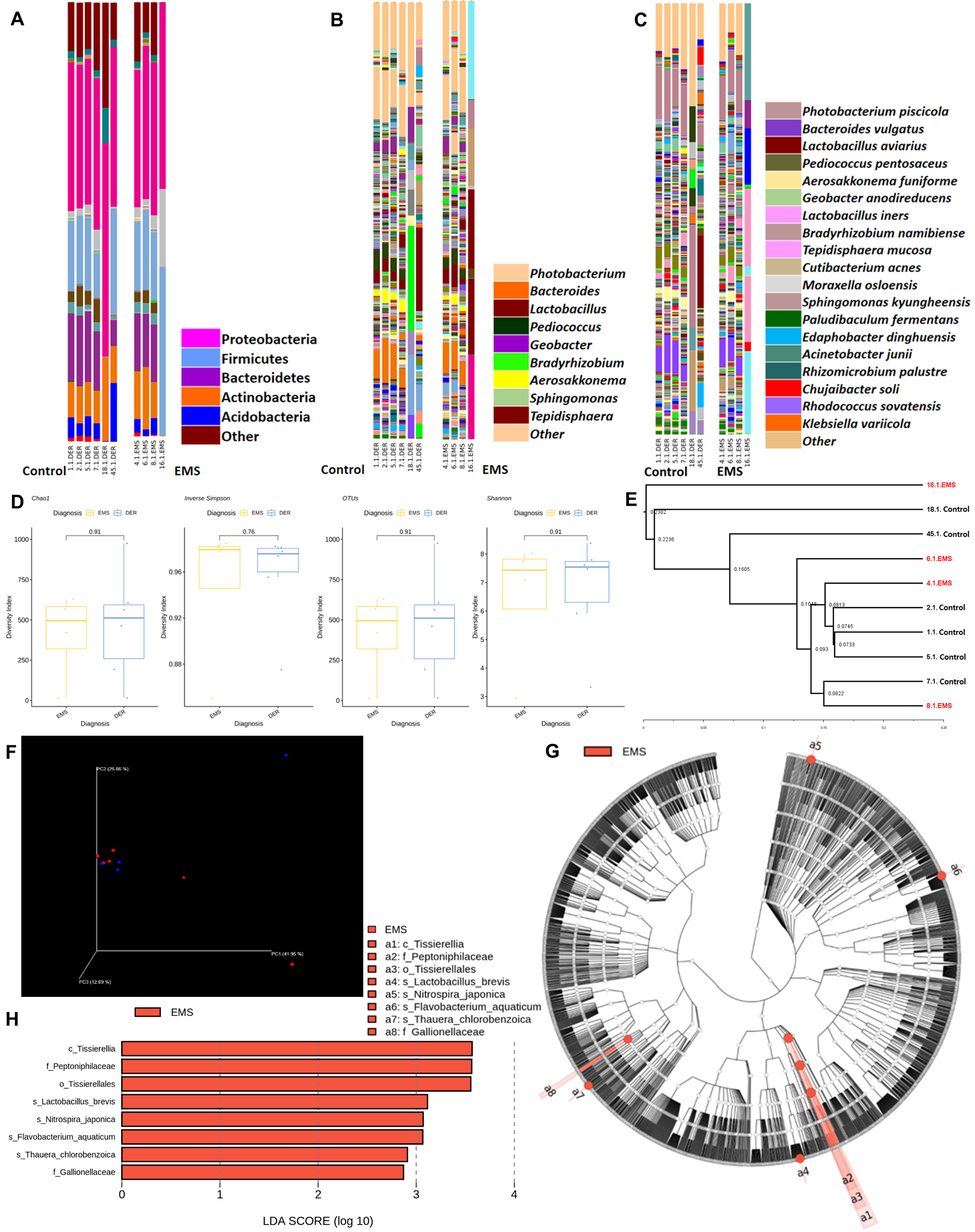

Supplement: Fig. S3 — Composition and differential analysis of the peritoneal microbiome in women with endometriosis and controls. [file spectrum.03689-25-s0003.tif]

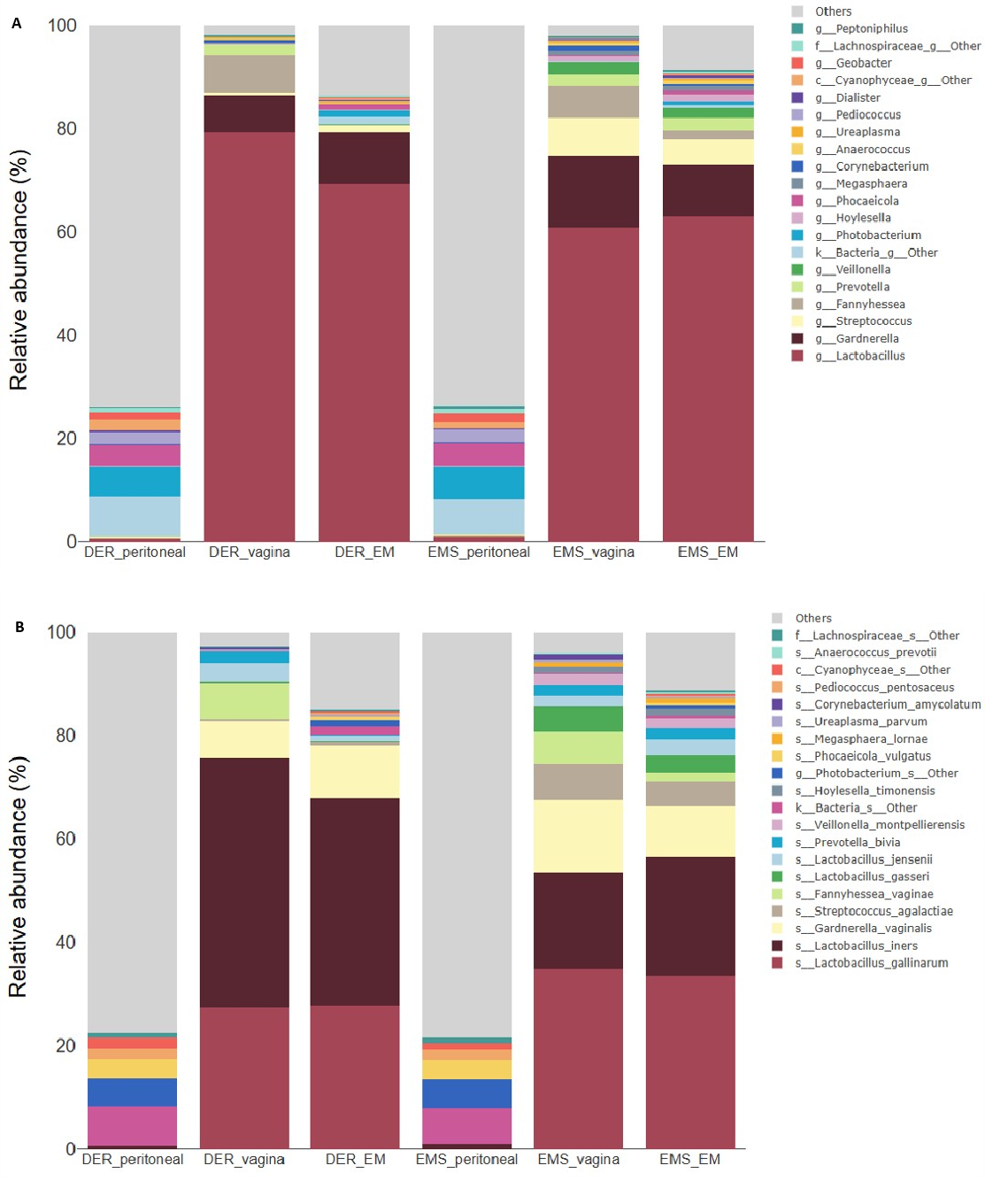

Supplement: Fig. S4 — ASV-based taxonomic composition of the genital tract microbiome at genus and species levels. [file spectrum.03689-25-s0004.tif]

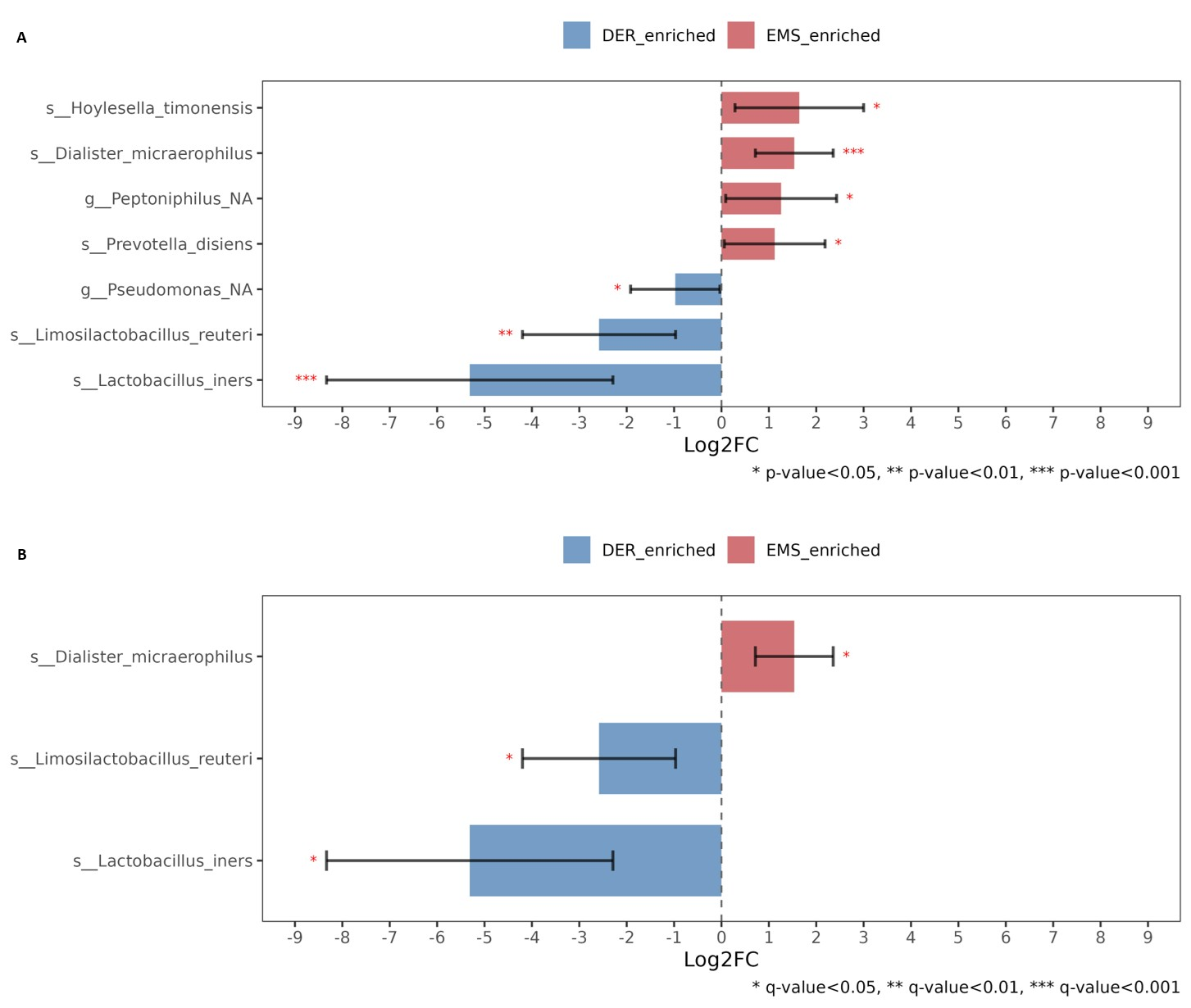

Supplement: Fig. S5 — ANCOM-BC-based differential abundance analysis of the vaginal microbiome in endometriosis and controls. [file spectrum.03689-25-s0005.tif]

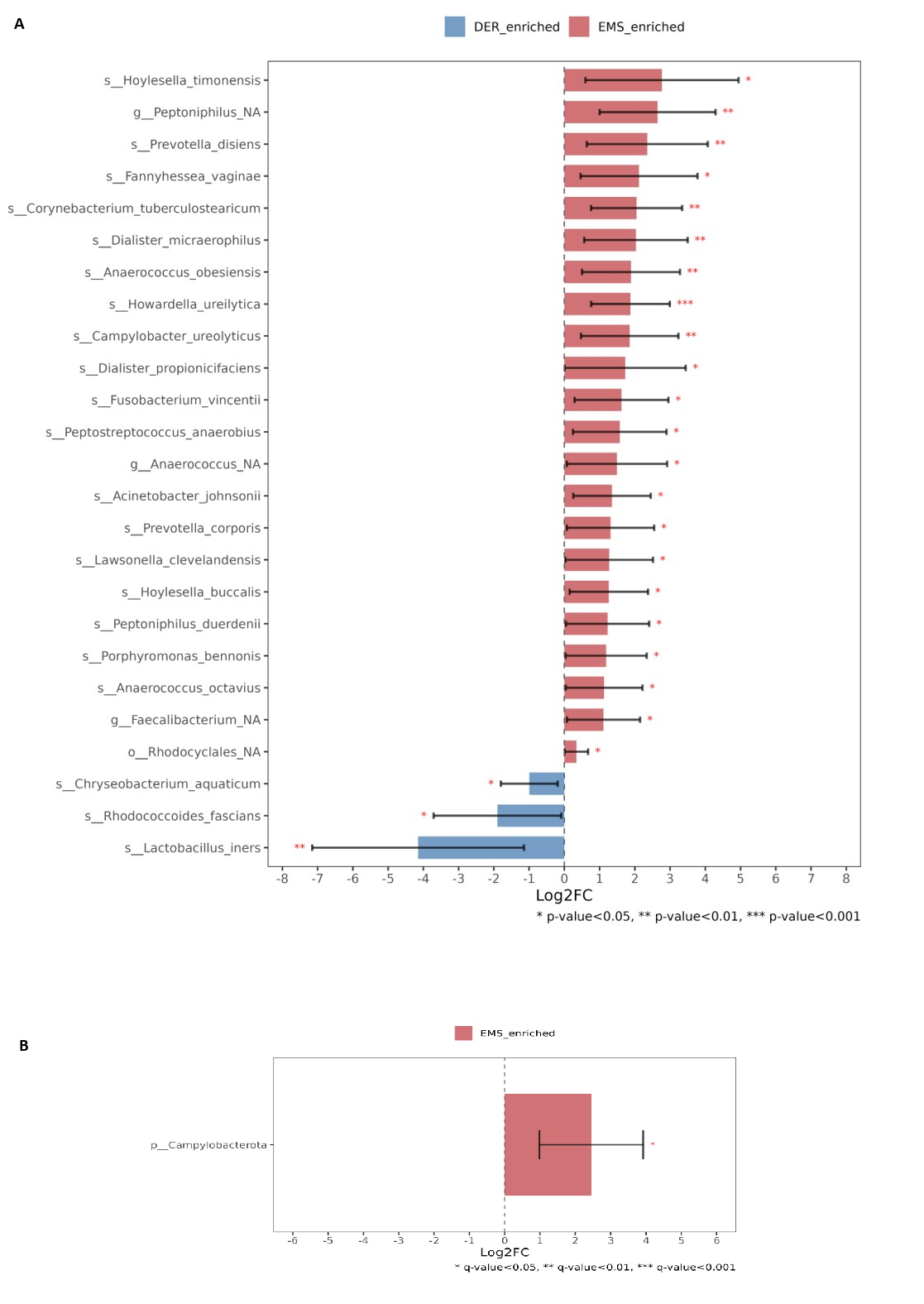

Supplement: Fig. S6 — ANCOM-BC-based differential abundance analysis of the endometrial microbiome in women with endometriosis and control subjects. [file spectrum.03689-25-s0006.tif]

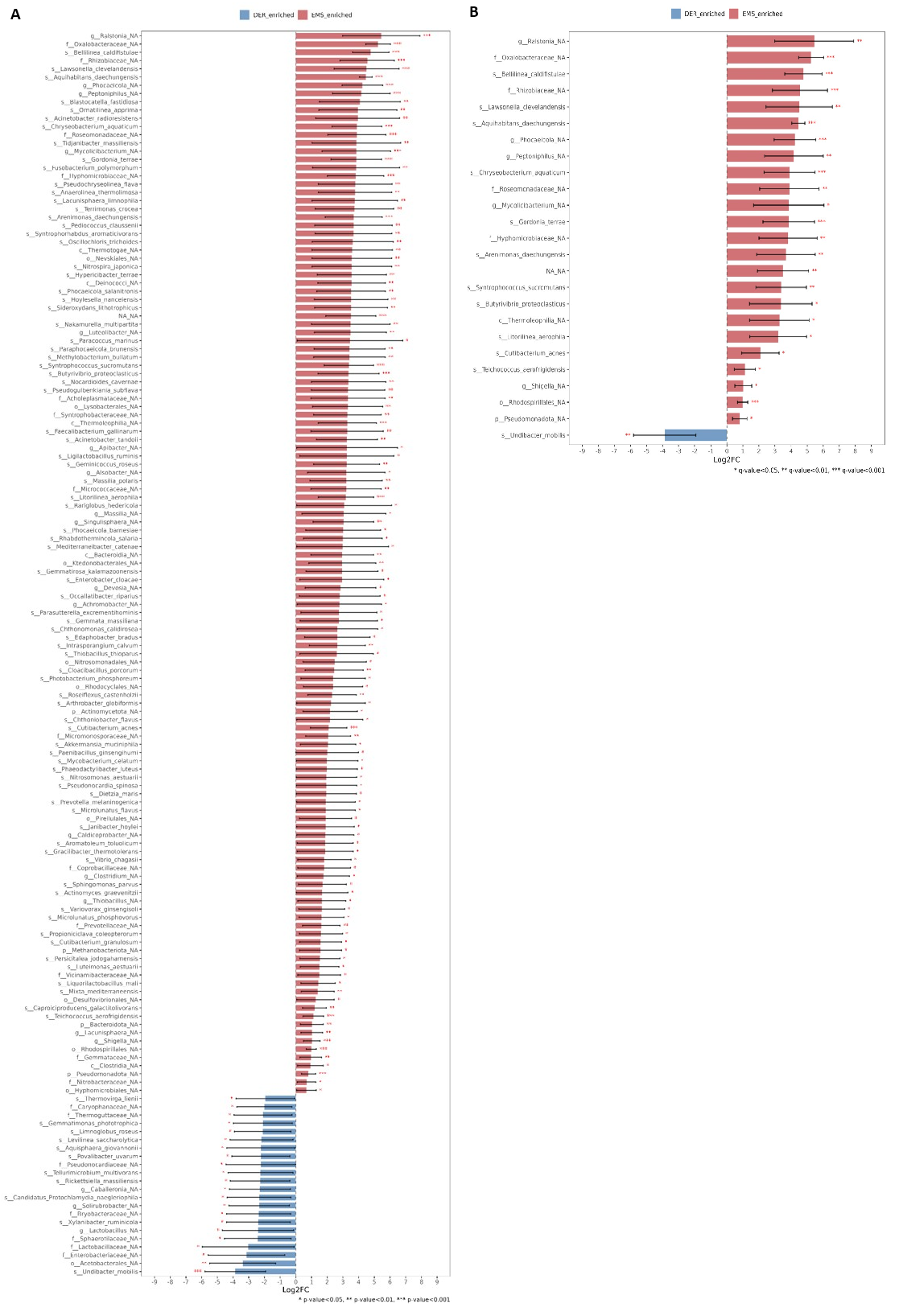

Supplement: Fig. S7 — ANCOM-BC-based differential abundance analysis of the peritoneal microbiome in endometriosis and control groups. [file spectrum.03689-25-s0007.tif]

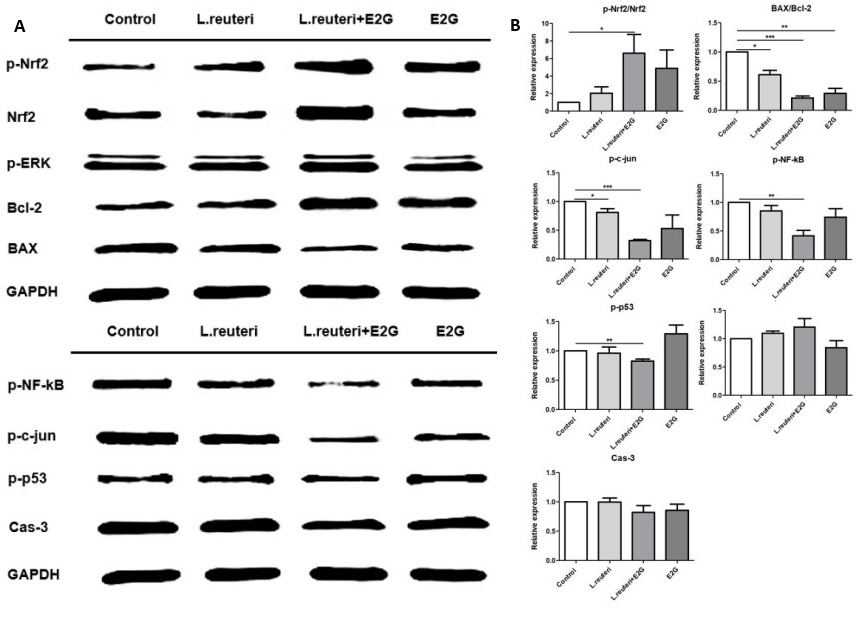

Supplement: Fig. S8 — Modulation of gene expression in endometrial cells under simulated follicular phase conditions (E2G + L. reuteri). [file spectrum.03689-25-s0008.tif]
