# Supplementary material for: Microbiome in women with endometriosis and the in vitro effects of Lactobacillus reuteri on human endometrium
Source: Microbiol Spectr. 2026 May 6;14(6):e03689-25. doi: 10.1128/spectrum.03689-25 (PMC13228028; doi:10.1128/spectrum.03689-25)
Supplement: Supplemental material — Table S1; Supplemental figure legends. [file spectrum.03689-25-s0009.docx]

**Supplementary Table**. Multiple reaction monitoring transitions for estrogen compounds.

| **#** | **Compound**  **name** | **Dansylated** | **Ion species** | **Precursor**  **ion** | | **Quantifier ion**  **(Collision energy, V)** | | **Qualifier ion**  **(Collision energy, V)** |
| --- | --- | --- | --- | --- | --- | --- | --- | --- |
| 1 | Estradiol-glucuronide | No | [M-H]- | 447 | 85 (26) | | 271 (30) | |
| 2 | Estradiol-^13^C_3_ | No | [M+H]+ | 509 | 171 (32) | | 156 (52) | |
| 3 | Estradiol | Yes | [M+H]+ | 506 | 171 (30) | | 156 (50) | |
| 4 | Estrone-^13^C_3_ | Yes | [M+H]+ | 507 | 171 (34) | | 156 (51) | |

**Legends for Supplementary Figures**

**Supplementary Figure 1.** Vaginal microbiome composition in women with endometriosis (EMS) compared to controls. Taxonomic profiles at the (A) phylum, (B) genus, and (C) species levels, showing predominance of Firmicutes, Lactobacillus, and Lactobacillus crispatus (*L. crispatus*), respectively. Although similar color shades are used for visualization*, L. crispatus* constitutes the predominant species across samples, whereas *Streptococcus agalactiae* and *Veillonella montpellierensis* represents only a minor fraction of the total relative abundance. Analyses of (D) α-diversity (Shannon, Inverse Simpson, Chao1) and (E, F) β-diversity (UPGMA, PCoA) revealed no significant differences between groups. (G–H) Differential abundance analyses (Wilcoxon test and LEfSe) identified taxa showing significant differences between EMS and control groups. The genus *Sutterella* and two species, *Dialister micraerophilus* and *Porphyromonas bennonis*, were enriched in the EMS group, whereas *L. iners* and *L. reuteri* were enriched in controls. (G) Cladogram of Linear discriminant analysis Effect Size (LEfSe) depicting taxonomic differences in the vaginal microbiome between EMS and control groups. Red nodes indicate taxa enriched in EMS, and blue nodes indicate taxa enriched in controls. Differentially abundant taxa included *Sutterella* (genus), Sutterellaceae (family), Bifidobacteriaceae (family), Bifidobacteriales (order), *Porphyromonas bennonis*, and *Dialister micraerophilus* in EMS, and *L. iners* and *L. reuteri* in controls. (H) Linear discriminant analysis (LDA) score plot highlighting the most discriminant taxa between groups (LDA score >2.0, p<0.05). Red bars represent taxa enriched in EMS, while blue bars represent taxa enriched in controls.

**Supplementary Figure 2.** Endometrial microbiome composition in women with endometriosis (EMS) and controls. (A–C) Taxonomic profiles at the phylum, genus, and species levels, showing predominance of Firmicutes, Lactobacillus, and Lactobacillus iners (L. iners), respectively. Although similar color shades are used for visualization*, L. crispatus* constitutes the predominant species across samples, whereas *Streptococcus agalactiae* and *Veillonella montpellierensis* represents only a minor fraction of the total relative abundance. (D–F) α-diversity (Shannon, Inverse Simpson, Chao1) and β-diversity (UPGMA, PCoA) analyses revealed no significant group differences. (G–H) Differential abundance analyses (Wilcoxon and LEfSe) identified enrichment of Campylobacter ureolyticus, Corynebacterium tuberculostearicum, Howardella ureilytica, and Prevotella timonensis in EMS, while 28 species, including Chryseobacterium greenlandense, L. reuteri, and Rhodanobacter glycinis, were more abundant in controls. (G) Cladogram of Linear discriminant analysis Effect Size (LEfSe) analysis showing taxonomic differences in the endometrial microbiome between EMS (red) and control (blue) groups. Red nodes represent taxa enriched in EMS, while blue nodes represent taxa enriched in controls. Enriched taxa in EMS included Prevotella timonensis, Campylobacter ureolyticus, Corynebacterium tuberculostearicum, and Howardella ureilytica. In contrast, multiple taxa—including Novosphingobium, Chryseobacterium, Cloacibacterium, Rhodanobacter, Smithella, Afifella, Pelobacter, Actinomadura, and others—were enriched in controls. (H) Linear discriminant analysis (LDA) score plot (log10) highlighting discriminant taxa (LDA score >2.0, p<0.05). Blue bars indicate taxa enriched in controls, and red bars indicate taxa enriched in EMS.

**Supplementary Figure 3. Composition and differential analysis of the peritoneal microbiome in women with endometriosis (EMS) and controls.** (A–C) Taxonomic profiles at the phylum, genus, and species levels, showing predominance of Proteobacteria, Photobacterium, and Photobacterium piscicola, respectively. (D–F) α-diversity indices (Shannon, Inverse Simpson, Chao1) and β-diversity analyses (UPGMA and PCoA) revealed no significant group differences. (G) of Linear discriminant analysis Effect Size (LEfSe) analysis showing taxonomic differences in the peritoneal microbiome between EMS and controls. Red nodes indicate taxa significantly enriched in EMS. Enriched taxa in EMS included Lactobacillus brevis, Nitrospira japonica, Flavobacterium aquaticum, and Thauera chlorobenzoica, along with higher-level taxa such as class Tissierellia, order Tissierellales, family Peptoniphilaceae, and family Gallionellaceae. (H) Linear discriminant analysis (LDA) score plot (log10) highlighting discriminant taxa (LDA score >2.0, p<0.05). All significant taxa were enriched in the EMS group.

**Supplementary Figure 4.** **ASV-based taxonomic composition of the genital tract microbiome at genus and species levels.** (A) Genus-level and (B) species-level relative abundance across peritoneal fluid, vagina, and endometrium in DER (control) and EMS groups. Values represent mean relative abundance (%). Low-abundance taxa (<1%) are grouped as “Others.

**Supplementary Figure 5. ANCOM-BC-based differential abundance analysis of the vaginal microbiome in endometriosis (EMS) and controls (DER).** (A) Taxa meeting raw p-value thresholds. (B) Taxa remaining significant after FDR correction (q-value). Bars indicate log2 fold change (EMS vs DER). Positive values denote enrichment in EMS; negative values denote enrichment in controls. Statistical significance is indicated as follows: *p* < 0.05, p < 0.01, *p* < 0.001 for panel (A); and *q* < 0.05, q < 0.01, *q* < 0.001 for panel (B).

**Supplementary Figure 6. ANCOM-BC-based differential abundance analysis of the endometrial microbiome in women with endometriosis (EMS) and control subjects (DER).** (A) Taxa meeting raw p-value thresholds. (B) Taxa remaining significant after FDR correction (q-value). Bars indicate log2 fold change (EMS vs DER); positive values denote EMS enrichment. Statistical significance is indicated as follows: *p* < 0.05, p < 0.01, *p* < 0.001 for panel (A); and *q* < 0.05, q < 0.01, *q* < 0.001 for panel (B).

**Supplementary Figure 7. ANCOM-BC-based differential abundance analysis of the peritoneal microbiome in endometriosis (EMS) and control (DER) groups.** (A) Differential taxa identified using unadjusted *p*-values. Red bars indicate taxa enriched in EMS, and blue bars indicate taxa enriched in controls. Horizontal bars represent log₂ fold change with error bars showing variability estimates. (B) Differential taxa identified after multiple-testing correction using *q*-values (FDR-adjusted). Only taxa remaining significant after correction are displayed. Color coding and effect size representation are as described for panel A. Statistical significance is indicated as follows: *p* < 0.05, p < 0.01, *p* < 0.001 for panel (A); and *q* < 0.05, q < 0.01, *q* < 0.001 for panel (B).

**Supplementary Figure 8. Modulation of gene expression in endometrial cells under simulated follicular phase conditions (E2G + *L. reuteri*).** (A, B) Protein expression of endometriosis-associated genes in endometrial cells after 24 h co-culture with *L. reuteri* (MOI = 0.5) and estradiol-17-glucuronide (E2G, 10 mM). Data are shown as mean ± SEM (N = 5). P(*) < 0.05; p(**) < 0.01; p(***) < 0.001.
